# An overview on reproductive isolation in *Oryza sativa* complex

**DOI:** 10.1093/aobpla/ply060

**Published:** 2018-11-22

**Authors:** Sadia Nadir, Sehroon Khan, Qian Zhu, Doku Henry, Li Wei, Dong Sun Lee, LiJuan Chen

**Affiliations:** 1 Rice Research Institute, Yunnan Agriculture University, Kunming, Yunnan, China; 2 Department of Chemistry, University of Science and Technology, Bannu, Khyber Pakhtunkhwa, Pakistan; 3 Centre for Mountain Ecosystem Studies, Kunming Institute of Botany, Chinese Academy of Sciences, Kunming, Yunnan, China; 4 Key Laboratory of Economic Plants and Biotechnology, Kunming Institute of Botany, Chinese Academy of Sciences, Kunming, Yunnan, China; 5 Biotechnology Lab Complex, CSIR-Crops Research Institute, Ghana; 6 State Key Laboratory for Conservation and Utilization of Bio-Resources in Yunnan, Yunnan Agricultural University, Kunming, Yunnan, China

**Keywords:** Divergent evolution, hybrids, *Oryza*, reproductive barrier

## Abstract

Reproductive isolation is generally regarded as the essence of the speciation process. Studying closely related species is convenient for understanding the genetic basis of reproductive isolation. Therefore, the present review is restricted to the species and subspecies of the *Oryza sativa* complex, which includes the two domestic rice cultivars and six wild species. Although closely related, these rice species are separated from each other by a range reproductive barriers. This review presents a comprehensive understanding of the forces that shaped the formation of reproductive barriers among and between the species of the *O. sativa* complex. We suggest the possibility that domestication and artificial breeding in these rice species can lead to the early stages of speciation. Understanding the evolutionary and molecular mechanisms underlying reproductive isolation in rice will increase our knowledge in speciation and would also offer practical significance for the implementation of crop improvement strategies.

## Teaser

This review presents a comprehensive understanding of the forces that shaped the formation of reproductive barriers among and between the species of the *Oryza sativa* complex.

## Introduction

Genetic divergence, reproductive isolation, natural selection and human-assisted artificial speciation are the vital forces that shape population genetics and consequent speciation (Liu *et al.* 2015; Schulter and Panell 2017). Reproductive isolation is a very important evolutionary phenomena that prevents genome homogenizations and maintains the integrity of species (Bomblies and Weigel 2007; Ouyang and Zhang 2013; Chen *et al.* 2016). The evolution of reproductive isolation allows differentiation and local adaptations to become fixed in diverging populations.

Rice belongs to the genus *Oryza*, which contains 25 recognized species, of which 23 are wild and 2 are domesticated (Vaughan *et al.* 2003). The genus *Oryza* has been classified into different species complexes based on their nine distinct genomes, viz., A, B, C, D, E, F, G, H and J (Vaughan *et al.* 2003). The *O. sativa* complex belongs to the A genome and contains two domesticated species *O. sativa* and *O. glaberrima* and six wild species: *O. rufipogon*, *O. nivara*, *O. barthii*, *O. longistaminata*, *O. meridionalis* and *O. glumaepatula*. These species constitute the primary gene pool of rice (Vaughan *et al.* 2003; Tripathi *et al.* 2011). The wild rice *O. rufipogon* is a perennial, outcrossing species widely distributed in Asia and Oceania. *Oryza nivara* is an annual, self-fertilized wild rice species mainly found in South and Southeast Asia. *Oryza rufipogon* and *O. nivara* are sometimes considered to be separate species or ecotypes of the same species (Sang and Ge 2007; Vaughan *et al.* 2008). The perennial *O. longistaminata* and the annual *O. barthii* (also called *O. breviligulata*) are the African wild rice species and can be found growing in the same area. *Oryza longistaminata* is a rhizomatous and self-incompatible species found to be the most diverged of all the species in the *O. sativa* complex (Vaughan *et al.* 2008). The annual *O. glumaepatula* is widespread in Tropical America, whereas the annual, inbreeding and highly diverged *O. meridionalis* is endemic to Tropical Australia and is often sympatric with *Oryza australiensis* (EE genome) in Australia. *Oryza sativa* is the Asian cultivated rice and is distributed globally, whereas *O. glaberrima* is the African cultivated rice and is mostly confined to Africa and differs from *O. sativa* in its morphology and ecology. *Oryza sativa* has been further subdivided into multiple varietal groups, the major ones being *indica* and *japonica* (Garris *et al.* 2005; Sweeney *et al.* 2007). In addition, weedy rice (*Oryza sativa* f*. spontanea*), which is conspecific and congeneric to cultivated rice, occurs together with cultivated rice in and around the rice fields (Nadir *et al.* 2017). The weedy rice associated with *O. sativa* may be called *O. sativa*, although they are not the crop. Those associated with *O. glaberrima* were sometimes called *O. stapfii* (Suh 2008).

**Figure 1.**
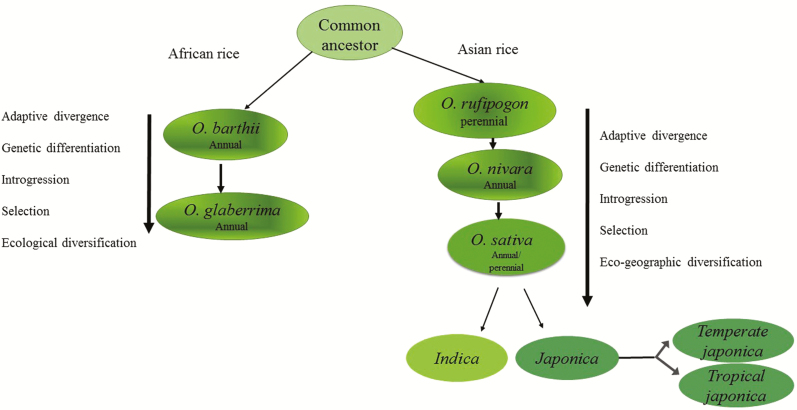
Simplified schematic representation of the evolutionary pathways of Asian and African cultivated rice and the evolutionary dynamics of reproductive barriers in rice. Strong selection during the domestication process, mutation accumulation, adaptation to different environments and diversification are the key factors in the evolution of reproductive barriers between rice populations.

The earliest evidence for the domestication of Asian rice, *O. sativa* found to date was at the region of the Yangtze River valley of China dated back to 11000–12000 BC (Vaughan *et al.* 2008; Gross and Zhao 2014). The wild *Oryza* species, *O. rufipogon* or *O. nivara* or possibly both of them, are the progenitors of *O. sativa* (Fig. 1) (Sang and Ge 2007; Vaughan *et al.* 2008). Apparently, conflicting data are available supporting single and multiple events leading to domestication of *O. sativa* (Oka 1988; Cheng *et al.* 2003; Londo *et al.* 2006; Vaughan *et al.* 2008; Molina *et al.* 2011; Huang *et al.* 2012). Molecular studies based on similarity in the alleles for non-shattering grains and erect growth in *indica* and *japonica* subspecies support the hypothesis of single domestication event (Li *et al.* 2006; Lin *et al.* 2007; Jin *et al.* 2008; Tan *et al.* 2008). These studies suggest that after domestication *O. sativa* spread and diversified to create divergent subgroups. Other studies based on the biochemical traits, hybrid sterility and subsequently supported by molecular analyses (Cheng *et al.* 2003; Tripathi *et al.* 2011) suggest that *indica* and *japonica* subspecies originated under separate domestication events from two divergent wild rice species in China and India, respectively (Tripathi *et al.* 2011). The African rice *O. glaberrima* was domesticated from *O. barthii* separately but parallel to the Asian rice in the African continent between 300 BC and 200 BC during a single domestication event (Fig. 1) (Murray 2004; Purugganan 2014). *Oryza barthii* was introduced from Asia into Africa (Vaughan *et al.* 2008).

Considerable high genetic variations exist in this primary gene pool of rice. For example, three regional variants of *O. glumaepatula*, five distinct groups of *O. longistaminata* and at least two different genetic groups in *O. rufipogon*, based on their ecology and life histories, have been recognized (Oka 1988; Akimoto *et al.* 1998; Vaughan *et al.* 2008). Similarly, the variation between *indica* and *japonica* subspecies of *O. sativa* is well documented (Garris *et al.* 2005). Differences were also recognized between temperate and tropical *japonica* varieties as well as within the tropical *japonica* varieties (Garris *et al.* 2005; Vaughan *et al.* 2008). Compared to *O. sativa*, *O. glaberrima* has a restricted geographic distribution and consequently lower genetic diversity exists in African rice (Semon *et al.* 2005). Only a few genetic subgroups have been detected in *O. glaberrima*, which reflects the ecological differentiation of *O. glaberrima* in different habitats (Semon *et al.* 2005).

**Figure 2.**
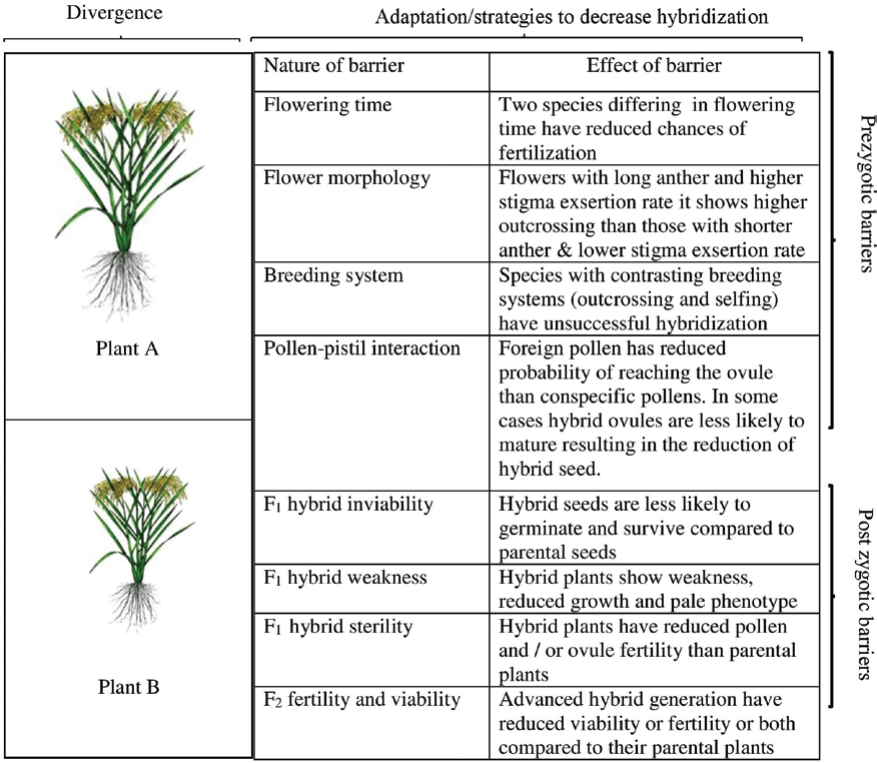
A schematic description of barriers contributing to reproductive isolation in rice. In this figure, (A) and (B) represent two diverged species. Divergence between species is associated with a set of morphological, developmental and genetic changes which create reproductive barriers between them and prevent them from breeding. Hybridization between two diverged species can result in maladapted, non-viable or infertile hybrids. Barriers are listed in the order in which they occur.

Almost all kinds of reproductive barriers so far reported in plants have been found in *O. sativa* complex and these include reduced cross-fertility, low germinability of F_1_ seeds, F_1_ inviability, F_1_ pollen and embryo sac sterility, and a sporophytic sterility and weakness in F_2_ generations also known as hybrid breakdown (Fig. 2) (Amemiya and Akemine 1963; Chu and Oka 1969, 1970; Ichtiani *et al.* 2007,^2011^; Chen *et al.* 2008,^2014^). Despite the occurrence of many barriers to hybridization, introgression among these *Oryza* species is common. Gene flow between *O. sativa* and *O. rufipogon* was identified in a number of studies (Pusadee *et al.* 2016; Wang *et al.* 2017). Gene flow has also been reported between *O. rufipogon* and *O. nivara* as well as between the *indica* and *japonica* subspecies of *O. sativa* (Zheng and Ge 2010; Yang *et al.* 2012). Hybridization between *O. sativa* and African wild and cultivated rice at varying level has also been reported (Jones *et al.* 1997).

Previously, comprehensive literature has been presented on the reproductive isolation in model as well as non-model organisms which has extending our understanding of reproductive isolation (Coyne and Orr 2004; Bombiles and Weigel 2007; Lowry *et al.* 2008; Ouyang and Zhang 2013; Baack *et al.* 2015). The topic of reproductive isolation in rice (*O. sativa*) has been reviewed in previous studies (Ouyang *et al.* 2010; Ouyang and Zhang 2013) but these studies have focused only on the hybrid sterility observed in *indica*–*japonica* hybridization. As rice is one of the better developed systems for understanding the evolution of reproductive isolation, we suggest that a broader view of the various factors that cause the reproductive isolation offers the opportunity to thoroughly understand the phenomena of reproductive isolation in rice. Here, we present a comprehensive study of closely related species of *O. sativa* complex and attempt to identify all reproductive barriers limiting hybridization. Understanding the molecular basis and the evolutionary forces that caused these barriers to evolve will increase our knowledge of reproductive isolation.

## Divergence Patterns and the Evolution of Reproductive Barriers in Rice

### Domestication: selection by early humans

Domestication is the result of a selection process by early farmers that led to the increased adaptation of a plant to cultivation and utilization by humans (Gross and Oslen 2010). The domestication process involves the repeated selection for desirable traits, resulting in the responsible gene mutations becoming fixed in the populations. In some cases, these mutations may lead to divergence and acquiring of variable degrees of isolation from their wild ancestors (Milla *et al.* 2015). After divergence, the domesticated species became dependent on humans for their reproduction and geographical spread (Milla *et al.* 2015).

Rice domestication under early human selection led to the intense morphological and physiological variations from its wild ancestors (Table 1) (Sweeney and McCouch 2007; Asano *et al.* 2011). These alterations resulted in the creation of high-yielding, uniform-germinating and densely planting present-day cultivated rice varieties. The most striking impact of domestication, which differentiated wild and cultivar populations into different reproductive and ecological realms, was changing rice from an outcrossing to an inbreeding crop under human selection for uniform traits. The wild species *O. rufipogon* and *O. barthii* are outcrossing, while *O. sativa* and *O. glaberrima* are almost entirely self-pollinated.

**Table 1.** Morphological changes associated with domestication in rice.

Trait	Wild rice	Domestic rice
Plant height	Tall	Medium to short
Growth habit	Creeping	Erect
Tiller number	Multiple spreading tillers	Reduced tillers
Breeding system	Outbreeding	Self-fertilized
Yield	Low	High
Seed quality	Non-glutinous	Glutinous
Seed dormancy	High seed dormancy	Low seed dormancy
Seed shattering	High shattering	Non-shattering
Floral structure	Long anthers long stigma	Short anthers short stigma
Panicle shape	Open panicle	Closed panicle
Grain size	Small	Variable
Awns	Long awns	Short awns
Hulls	Dark/black coloured	Straw coloured
Pericarp/seed coat	Pigmented	Most Asian cultivars lack pigmentation, but many African cultivars retain

**Figure 3.**
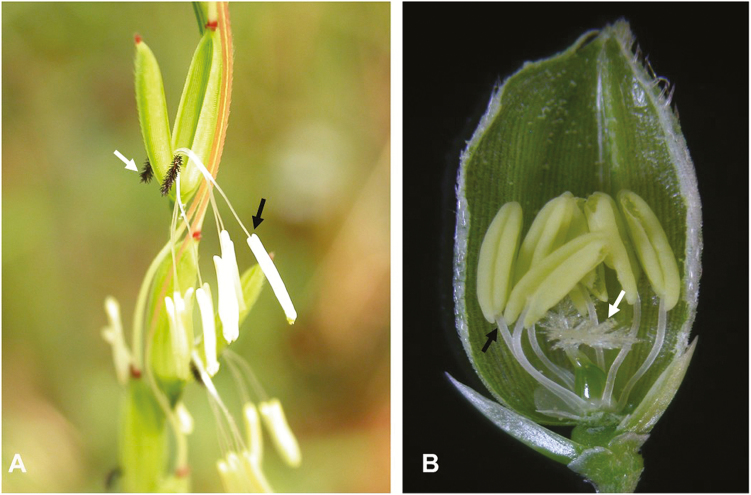
Wild and cultivated rice phenotypes. (A) and (B) represent comparative phenotypes of wild and cultivar open florets. Black arrows indicate anthers and white arrows indicate stigmas.

*Oryza sativa* has a limited degree of outcrossing owing to the short style and stigma, short anthers, limited pollen viability and the brief period between flower opening and pollen release (Tripatti *et al.* 2011). However, the wild ancestor *O. rufipogon* have large stigma, and long anthers (Fig. 3). The shift to selfing is associated with changes in flower morphology, matting patterns and reproductive investment that can in turn affect the extent of hybridization and gene flow between populations (Wendt *et al.* 2002; Coyne and Orr 2004; Martin and Willis 2007; Sicard and Lenhard 2011; Wright *et al.* 2013). This suggests that different suites of genes and the corresponding positive mutations that accumulated in the domesticated genotype would be absent from the wild genotype. Therefore, when crosses are made between the domestic and wild populations, domestication-related loci may not interact well with each other.

### Crop improvement: selection by breeders

Crop improvement involves the incorporation of as many desirable characteristics as possible into a single variety to make it a superior variety. Rice crop improvement tends to produce new varieties with increased yields, enhanced grain characteristics and nutritional values, and an increased resistance against various biotic and abiotic factors. Selective breeding is also associated with the spreading of genes among/or between populations (Chen *et al.* 2016). As an example, the domestication allele of the reduced shattering gene *sh4* in rice originated in the *japonica* group and spread to the *indica* group through selective breeding (Gross and Olsen 2010). Selective breeding favours the white grains of domesticated varieties compared with the pigmented grains of wild rice. The gene *OsC1* is responsible for the grain pigmentation (Saitoh *et al.* 2004). *OsC1* is tightly linked with the sterility locus *S5* (Saitoh *et al.* 2004). *S5* is a major locus controlling embryo-sac fertility and is responsible for the low fertility observed in the *indica*–*japonica* hybridizations. The *S5* locus contains three alleles: an *indica* allele (*S5i*), *japonica* allele (*S5j*) and a neutral allele (*S5n*) (Chen *et al.* 2008). *S5i* and *S5j* are each compatible with *S5n*, but the combination of *S5i* and *S5j* leads to hybrid sterility. Independent mutations to *OsC1* leading to lighter grain colour occurred on the background of *S5i* and *S5j*, respectively. Selection for the light-colour *OsC1* allele indirectly led to increased frequencies of the *S5i* and *S5j* alleles at the *S5* locus. Thus, selective breeding for *OsC1* results in the increase in frequency of *S5* genes in the population and an increase in sterility barrier which indicates that *S5* locus and post-zygotic isolation can arose as a by-product of genetic hitchhiking between tightly linked genes in rice (Saitoh *et al.* 2004). Another well-documented example results from the tight linkage between the yield establishment gene *Gna1* and the sterility locus *S35*. Again, selection for the yield increasing gene might have indirectly favoured the retention of a sterility locus in the *indica* and *japonica* subspecies (Chen *et al.* 2016; Kubo *et al.* 2016). Generally, the wild relatives of rice crops are an important reservoir of genetic variability for various economic characteristics, such as disease and insect resistance, tolerance to abiotic stresses, male sterility, increased biomass, grain yield and improved quality characteristics (Xiao *et al.* 1998). However, the hybridization between cultivated and wild species is often limited by linkages between desirable and undesirable genes that may hinder the production of lines with desirable agronomic characteristics (Xiao *et al.* 1998). For example, many desirable agronomic traits in wild rice are often linked with the easy shattering phenotype (Xiao *et al.* 1998). This suggests that linkage between favourable and unfavourable alleles may limit introgression.

## Reproductive Barriers in Rice

### Pre-zygotic reproductive barriers

In some well-studied cases of plants, pre-zygotic reproductive barriers are found to be stronger and lead to nearly complete reproductive isolation (Lowry *et al.* 2008). The adaptation of species or subspecies to different environments has been recognized as an important reproductive isolating barrier (Baack *et al.* 2015). Local adaptation involves the evolution of traits in response to different environmental conditions (Baack *et al.* 2015). These traits include abiotic stress tolerance, breeding times, changes in floral characteristics, flowering time and gametes compatibility that can create reproductive incompatibility between populations (Baack *et al.* 2015). The sympatrically growing species *O. barthii*–*O. glaberrima* and *O. nivara*–*O. rufipogon* are precluded from hybridization by the differences in flowering time (Zheng and Ge 2010; Liu *et al.* 2015). Incompatibility in pollen–pistil interactions provides a strong pre-zygotic reproductive barrier (Oka 1988; Harushima *et al.* 2002; Lowry *et al.* 2008). Crosses between *O. nivara*–*O. sativa* and *O. breviligulata*–*O. glaberrima* showed embryo sac sterility (Chu and Oka 1970).

### Post-zygotic reproductive barriers

Post-zygotic reproductive barriers preclude the development of viable fertile hybrid progeny. These barriers in rice begin at hybrid seed development and continue until the hybrid plant reaches the seed-producing stage (Tiffin *et al.* 2001). At each developmental stage in a plant’s life cycle, a barrier prevents the hybrid progeny from reaching the next developmental stage. These post-zygotic barriers are further classified based on the stage of occurrence.

### Hybrid inviability: an endosperm-based hybridization barrier

The endosperm is the basic nutrient source for the developing embryo (Lafon-Placette and Köhler 2016). Any abnormality during endosperm development ultimately leads to embryo arrest and seed failure (Lafon-Placette and Köhler 2016). In dicot species like *Arabidopsis*, the endosperm supports embryonic growth and disappears soon after cellularization (Bushell *et al.* 2003). In monocot species, like rice, the endosperm continues to proliferate and support seedling growth even after germination (Sekine *et al.* 2013). Seeds that lack properly developed endosperm fail to germinate. The endosperm is a highly sensitive tissue and requires a relative maternal to paternal genome dosage ratio of 2:1 for successful development and requires a highly specific balanced gene expression (Lafon-Placette and Köhler 2016). Defects in parental genome dosages, caused by either interspecific crosses or interploidy crosses, are the main reasons for hybrid endosperm defects (Ishikawa *et al.* 2011; Sekine *et al.* 2013). Genomic imprinting, whereby some genes are expressed in parental origin-specific manners, serves as the molecular basis for parental genome dosage effects (Matsubara *et al.* 2003; Ishikawa *et al.* 2011; Chen *et al.* 2016).

Generally, outbreeding is thought to increase the intensity levels of the parental genome conflict and the associated genomic imprinting, while inbreeding is thought to reduce those (Lafon-Placette and Köhler 2016). Interspecific hybridizations between *O. rufipogon* × *O. sativa* and *O. longistamanita* × *O. sativa* resulted in dosage imbalance in the hybrid endosperm leading to its developmental defects (Matsubara *et al.* 2003; Ishikawa *et al.* 2011). Similar endosperm development defects were observed in F_1_ embryos obtained from *O. barthii* × *O. sativa*, *O. barthii* × *O. glaberrima* hybridizations (Chu and Oka 1970). Parental genome conflicts arising from ploidy variations have also been reported in a cross between diploid and tetraploid *japonica* rice (Sekine *et al.* 2013). Endosperm-based hybridization barriers are the evolutionary forces associated with parental genome conflict, which might arose as a result of a shift in mating processes (i.e. from outbreeding to inbreeding) and thus established a barrier to interploidy and interspecific hybridization in rice.

### Hybrid weakness: a post-embryonic-stage hybridization barrier

Hybrid weakness also known as hybrid necrosis is a post-embryonic-stage barrier frequently observed in plant taxa (Bomblies and Weigel 2007). The phenotype of hybrid weakness is similar to the symptoms associated with disease responses (Bomblies and Weigel 2007). Hybrid weakness has been reported in many crosses involving *O. rufipogon* × *O. sativa*, *O. barthii* × *O. glaberrima* as well as between the subspecies of *O. sativa* (*indica* × *indica* and *japonica* × *japonica* hybridizations) (Ichitani *et al.* 2011; Zhang *et al.* 2012; Chen *et al.* 2013, 2014). Early embryogenesis is normal, and normal seedlings are established (Chen *et al.* 2013). However, at later stages, the seedlings fail to grow properly. The hybrid seedlings have retarded growth rates, with a pale yellow phenotype, and they undergo wilting and necrosis (Chen *et al.* 2013, 2014). Although, many cases of hybrid weakness have been reported in rice, the exact molecular mechanism underlying hybrid weakness is still not well known. Previous studies have suggested that hybrid weakness is under the control of complementary gene either dominant or recessive (Amemiya and Akemine 1963; Chu and Oka 1970; Ichitani *et al.* 2007, 2011; Saito *et al.* 2007; Kuboyama *et al.* 2009; Chen *et al.* 2014). Some studies have suggested that hybrid weakness is due to the activity of defence-related genes which was further confirmed by the high activity of F_1_ hybrids against pathogens (Bombiles and Weigel 2007; Chen *et al.* 2014). Recently, genes conferring hybrid weakness in *O. rufipogon* × *O. sativa* F_1_ hybrids have been cloned (Chen *et al.* 2014). Molecular studies identifying the casual gene of hybrid weakness will help in detailed understanding of the phenomena.

### Hybrid sterility: a reproductive-stage hybridization barrier

Hybrid sterility is the most common form of post-zygotic hybridization barrier in plants (Ouyang *et al.* 2010). A well-documented example is the hybrid sterility observed in crosses between *O. sativa* and *O. meridionalis*, *O. sativa* and *O. glaberrima* and between *indica* and *japonica* subspecies of *O. sativa* (Ouyang *et al.* 2010; Yu *et al.* 2018). The hybrids obtained are robust in their vegetative growth; however, the progeny are often sterile and cannot produce the next generation. The physiological determinants of reproductive failure in rice include female gamete abortion, pollen sterility, reduced affinity between the uniting gametes, panicle growth rate and ovary growth (Chen *et al.* 2008; Long *et al.* 2008; Mizuta *et al.* 2010). Approximately 50 loci have been identified as being involved in the control of *indica–japonica* hybrid sterility (Chen *et al.* 2008; Long *et al.* 2008; Yamagata *et al.* 2010; Yang *et al.* 2012a; Kubo *et al.* 2016; Li *et al.* 2017). Several sterility loci have been identified and mapped as a single genetic loci in *O. glaberrima* (Hu *et al.* 2006; Li *et al.* 2011). Further studies relating to gene characterization will help in understanding the molecular mechanism underlying hybrid sterility. Recently, Yu *et al.* (2018) mapped a sterility locus that contains two tightly linked open reading frames (ORFs) that confers hybrid sterility in F_1_ hybrids derived from crossing *O. sativa* × *O. meridionalis*. One of the ORFs encodes a toxin, which affects the development of pollen, and the other encodes an antidote, which is required for pollen viability. Hybrid breakdown is the weakness and sporophytic sterility found in the F_2_ and advanced generations, and can be genetically different from F_1_ weakness or sterility (Okuno and Fukuoka 1999). Hybrid breakdown has been detected in many crosses of rice (Okuno and Fukuoka 1999).

## Genetic Models for the Evolution of Reproductive Barriers in Rice

Three genetic models have been developed to explain the kinds of genetic changes that occur to cause reproductive isolation in rice.

### Complementary epistasis interaction between two loci

**Figure 4.**
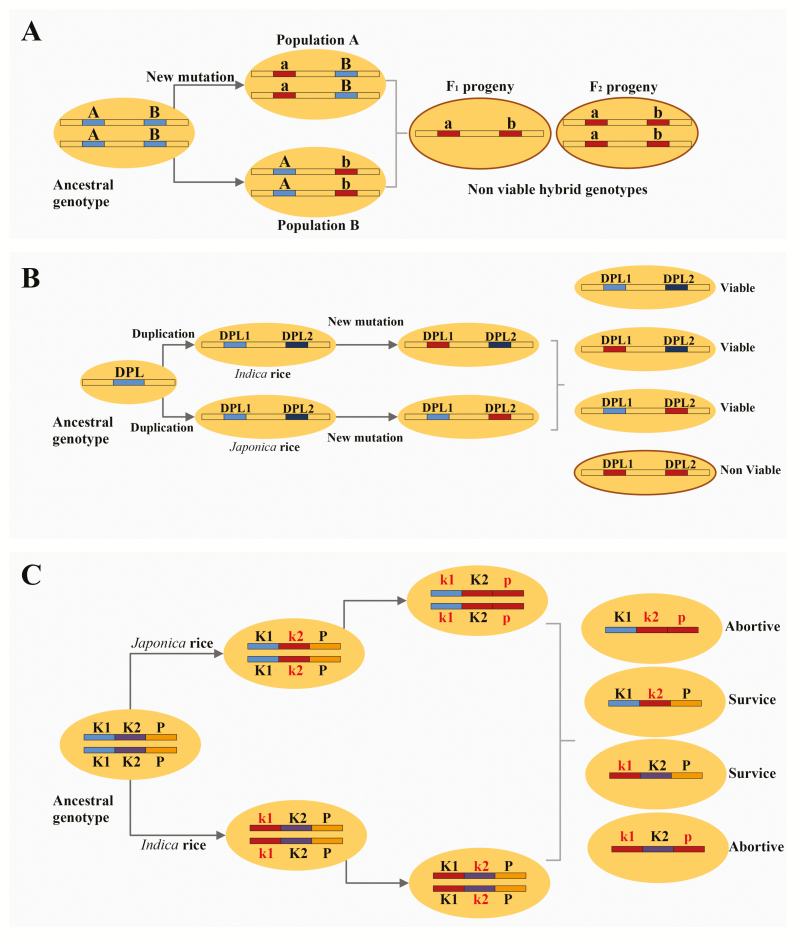
Genetic models for the evolution of reproductive isolation in rice. (A) A two-locus interaction in post-zygotic reproductive isolation in rice. An ancestral population splits into isolated populations that diverge genetically as a result of fixation of an independent mutation at each locus. If the two diverged populations hybridize, 1 in 4 F_1_ hybrids and 1 in 16 F_2_ progeny will have incompatible genotypes. (B) Gene duplication in post-zygotic reproductive isolation in rice. An ancestral population undergoes a duplication event followed by divergence due to a mutation (either a gain of deleterious function or loss of function). In the F_1_ hybrid, these two mutated alleles are incompatible (Mizuta *et al.* 2010). (C) A simple illustration for three tightly linked genes at the *S5* locus. K1 and K2 represent Killer genes, and P represent the Protector gene. The haplotype K1K2P represents a balance between killing and protecting of the gametes according to the genetic model in the *S5* system. *Indica* and *japonica* rice have independent mutations in these three linked genes, with *japonica* haplotype being k_1_K2p and *indica* haplotype being K1k_2_P. The gametes carrying loss of protector (p) will be non-viable.

The simplest genetic explanation of the complementary epistatic interactions between two loci that lead to hybrid inferiority was proposed by Bateson, Dobzhansky and Muller and termed the BDM model (Coyne and Orr 2004). These two loci may be duplicate gene copies or the same locus evolving repeatedly in one species or differently in two species. Figure 4A show a model representation of two-locus interaction causing hybrid incompatibility. Reviewing the literature of hybrid incompatibility cases in rice, a two-locus interaction seems to be the most common cause. A two-locus interaction leading to hybrid weakness was recently reported in *O. rufipogon* and *O. sativa* (*indica*) hybridization (Chen *et al.* 2014). The hybrids were found to have elevated immune responses similar to previously reported in *Arabidopsis* (Bombiles and Weigel 2007). The genes in hybrid weakness were found to encode defence-related proteins against pathogens and that incompatible allelic combinations appear to induce autoimmune responses (Chen *et al.* 2014). Similar two-locus interaction between pathogen resistance genes and their interacting partners has been implicated in hybrid weakness between the *indica* and *japonica* subspecies of cultivated rice (Yamamoto *et al.* 2010).

### Differential silencing

Molecular divergence owing to the divergent evolution of duplicated genes has been identified as a potential source of reproductive isolation (Werth and Windham 1991). After the duplication event, it is highly probable that both or one of the genes will mutate (Prince and Pickett 2002). Therefore, it can be assumed that an independent mutational event causes a pair of paralogous genes to undergo divergent evolutionary paths and becomes fixed in the two diverging populations. When the two populations hybridize, the populations carrying either of the functional copies develop properly, whereas the hybrids receiving the two silenced copies will have reduced fitness levels. The loss of the duplicated gene that encodes an essential protein for pollen development causes pollen sterility in the F_1_ interspecific hybrids of *O. sativa* and *O. glumaepatula* (Yamagata *et al.* 2010). Additionally, the independent disruption of duplicated genes *DOPPELGANGER1* and *DOPPELGANGER2* in the *indica* and *japonica* cultivars, respectively, causes pollen sterility in inter-subspecific F_1_ hybrids (Fig. 4B) (Mizuta *et al.* 2010). Likewise, duplication and the reciprocal silencing due to loss-of-function mutation of duplicate genes *S27* and *S28*, encoding a mitochondrial protein, were observed in the F_1_ interspecific hybrids of *O. sativa* and *O. glumaepatula* (Yamagata *et al.* 2010). Thus, gene duplication and subsequent mutations may play important roles in establishing reproductive barriers in rice.

### Genic interactions

A well-documented example of genic interactions leading to hybrid sterility comes from the tight linkage of three genes at the *S5* locus causing female gamete abortion and hybrid sterility in *indica japonica* hybridization (Yang *et al.* 2012b). Two of these three genes constitute the killer system which will preferentially kill the gametes which lack the protector. The divergence in any of these three genes does not occur independently and the evolution of one gene is expected to be conditional on the evolution of another gene. Therefore, a non-functional mutation in the killer would not cause the loss-of-function mutation in the protector. Rather, once the loss-of-function mutation in the killer exists, loss-of-function mutation in the protector would no longer be deleterious and might drift to fixation. Subsequent evolution in the *indica* and *japonica* subspecies has silenced different parts of the killer/protector system and neither subspecies has a functional killer phenotype. In heterozygotes, different genotypes will be formed of these three-gene combinations, hybrid sterility will appear owing to the deleterious interaction between the killer loci without protection by the protector (Yang *et al.* 2012) (Fig. 4C). Similar findings have been reported by Yu *et al.* (2018) where a selfish genetic element that encodes for a toxin and antidote that affects pollen viability causes hybrid sterility in *O. meridionalis* and *O. sativa* hybrids.

## Introgression across Barriers

### Crop-to-weed gene flow

Weedy rice is one of the troublesome weeds that grows sympatrically with the cultivated rice in rice fields (Nadir *et al.* 2017). Variable level of gene flow has been recorded to occur between cultivated rice and weedy rice (Langevin *et al.* 1990; Gealy *et al.* 2003; Nadir *et al.* 2017). One study has reported that these two sympatric populations can hybridize with each other freely without any sterility issue which indicates that fewer post-zygotic barriers exist between them (Craig *et al.* 2014). Furthermore, gene flow in reverse order from weed to crop has also been reported (Burgos *et al.* 2008; Serrat *et al.* 2013). Gene flow in either direction may have detrimental environmental consequence such as the evolution of undesired agronomic characters in rice crop or the evolution of more aggressive weeds that are difficult to control.

### Crop-to-wild gene flow

Domesticated rice mate with wild relatives and variable level of gene flow has been reported to occur (Langevin *et al.* 1990; Majumder *et al.* 1997; Song *et al.* 2002). *Oryza sativa* frequently hybridize with *O. rufipogon*. The morphological differences in floral traits suggest that gene flow will predominantly be from *O. sativa* to *O. rufipogon* and the gene flow shows distinct geographic patterns and varies with the *O. sativa* subspecies (Wang *et al.* 2017). The gene flow from domestic to wild rice has the potential to affect the genetic structure of wild rice (Wang *et al.* 2017). Wild-to-crop gene flow can be expected even though the frequency is lower than that of crop-to-wild gene flow. The asymmetric gene flow recorded in the *O. rufipogon* and *O. sativa* should be further explored to gain an insight into the forces and mechanism that determine reproductive isolation. These data can also help us in understanding the patterns of population-specific reproductive isolation in rice.

### Crop-to-crop gene flow

The two domestic rice species *O. sativa* and *O. glaberrima* represent parallel domestications from different progenitors on two different continents assisted by nearly same set of genes (Purugganan 2014). This suggests a high level of homology between the genomes of these *Oryza* species and the possibility to carry out gene transfer between them but this is difficult because of strong reproductive barriers between them (Chu and Oka *et al.* 1969). Similarly, gene exchange between *indica* and *japonica* subspecies of *O. sativa* has also been reported (Yang *et al*. 2012b). Gene exchange between these rice subspecies would be highly beneficial to rice breeding practices, but the sterility barriers hinder the exchange (Chen *et al.* 2008).

In this condition, we may assume that a certain rate of gene exchange takes place across the complex of isolating barriers (Zheng and Ge 2010). Perhaps, a barrier protects populations from reproductive waste, whereas introgression across the barrier offers genetic variability to the populations, giving rise to a balance between isolation and hybridization (Chu *et al.* 1969). With the rapid advances in transgenic biotechnology, several transgenic crop varieties have been developed and released into markets (Kwit *et al.* 2011). Synthetic genotypes with desired properties have been developed in rice (Croughan 1998; Paine *et al.* 2005). Crop-to-weed or crop-to-wild gene flow is of special concern when transgenes are involved (Lu and Snow 2005). These transgenes can escape and introgress into the wild and weedy population with potentially serious consequences (Lu and Snow 2005). The development of genetically modified crops has been accompanied by efforts to development of intrinsic genetic barriers to prevent transgene flow between synthetic and wild/weedy populations. These include interfering with pollination and fertilization using maternal inheritance and male sterility, terminating transgenic fruit/seed development (seed sterility), selectively terminable transgenic lines or compromising the fitness of hybrids that have acquired positive survival traits from crop genes through introgression (transgenic mitigation) (Suh 2008; Kwit *et al.* 2011; Nadir *et al.* 2017). This may also have the side effect of constructing intrinsic barriers to gene flow between independently synthesized transgenic lines that originate from the same ancestral forms (Schluter and Pennel 2017).

## Future Research Perspective

Rice serves as an excellent model for understanding speciation, and some species (*O. rufipogon* and *O. nivara*) are in the early stages of divergence. Therefore, a comprehensive understanding of all the forces and mechanisms that drive reproductive isolation is required. Some additional studies needed in this field are listed below:

1. Analysis of the quantification of the strength of the individual barrier and its contribution to the reproductive isolation in rice would allow us to better evaluate the importance of gene flow and reproductive isolation.
2. Although species of different ploidy are generally reproductively isolated from each other, gene flow across ploidy barriers has been reported in many plant taxa (Chapman and Abbott 2010; Pinheiro *et al.* 2010). However, in rice introgression across ploidy barrier is not well studied. Understanding the gene flow in ploidy variation and its impact on the morphology and ecology of rice species will be helpful in understanding the diversification and evolution of rice.
3. In addition to the crop-wild (interspecific) and *indica*–*japonica* (inter-subspecific) reproductive barriers, some barriers also exist between intra-subspecific hybridizations (*indica*–*indica* and *japonica*– *japonica*) (Zhang 2012; Fu *et al.* 2013). The hybrid weakness observed in the intra-subspecific hybridization (*indica*–*indica* and *japonica*–*japonica*) needs evolutionary as well as molecular analysis.
4. Few genes involved in reproductive isolation in rice have been identified and cloned (Chen *et al.* 2008; Long *et al.* 2008; Chen *et al.* 2013, 2014; Yu *et al.* 2018). However, the biochemical and molecular mechanisms, and the relationships between the causal genes are still largely unknown. For a complete understanding of these processes, further efforts are needed to characterize the mechanisms that control how gene products function to induce hybrid dysfunction at the molecular, cellular and organ levels. These studies will not only improve our understanding of reproductive isolation but also help to improve crop breeding strategies.

## Conflict of Interest

None declared.

## Sources of Funding

This study was supported by a grant from the National Key Research and Development Program of China (grant no. 2017YFD0100205), a grant from National Natural Science Foundation of China (grant no. 31560115), a grant from Yunnan Key Research and Development (grant no. 2018ZG005) and Yunling Super-Talent Initiative-“Yunling High-End Foreign Expert” programme.
